# Third-Component-Regulated Choline Chloride–Monoethanolamine-Based Solvent Systems for Enhanced Valorization of Bamboo Toward Concurrent Bioethanol and Carbon Dot Production

**DOI:** 10.3390/molecules31111832

**Published:** 2026-05-26

**Authors:** Sicheng Jin, Yongan Meng, Dongtian Miao, Chun Shi, Jing Yang, Zhengjun Shi, Haiyan Yang

**Affiliations:** College of Material and Chemical Engineering, Southwest Forestry University, Kunming 650224, China

**Keywords:** bamboo, deep eutectic solvent, delignification, enzymatic saccharification, bioethanol

## Abstract

Efficient pretreatment is essential for improving the conversion of lignocellulose into fermentable sugars and bioethanol. In this study, choline chloride–monoethanolamine (ChCl-MEA)-based solvent systems containing H_2_O_2_, NaHCO_3_, Na_2_S, or ethylene glycol were prepared and applied for the pretreatment of *Dendrocalamus brandisii*. Among the tested systems, ChCl-MEA-Na_2_S showed the best overall pretreatment performance, achieving 92.8 ± 2.3% delignification and 86.1 ± 1.7% cellulose retention. It also effectively disrupted lignin–carbohydrate associations, reduced lignin shielding and generated a more accessible cellulose-rich substrate for bioconversion. In the following separation enzymatic hydrolysis and fermentation, 92.2 ± 2.2% cellulose in substrate was converted to glucose, and 17.49 ± 0.7 g/L ethanol was obtained via the fermentation of enzymatic hydrolysate. Taking the bioconversion of substrate into consideration, the ChCl-MEA-H_2_O_2_ and ChCl-MEA-Na_2_S were recovered for full component utilization. Especially, the carbon dots produced from the degradation compounds in ChCl-MEA-H_2_O_2_ DESs had favorable antioxidation and antibacterial performance due to the oxygen-containing group caused by oxidation of H_2_O_2_.

## 1. Introduction

The increasing depletion of fossil resources and the growing concern over environmental pollution have accelerated the search for renewable feedstocks for sustainable energy and chemical production [1]. Lignocellulosic biomass, as the most abundant renewable organic carbon resource on Earth, is mainly composed of cellulose, hemicellulose, and lignin, and has great potential for the production of biofuels, biochemicals, and advanced biomaterials [2]. However, the compact and recalcitrant cell-wall architecture of lignocellulose, which is governed by extensive hydrogen bonding, lignin–carbohydrate associations, and hydrophobic interactions, severely restricts its efficient fractionation and subsequent valorization [3]. Conventional pretreatment methods, such as dilute acid, alkali, and organic solvent treatments, can improve biomass deconstruction to some extent, but they often suffer from harsh operating conditions, equipment corrosion, solvent recovery burdens, and undesirable degradation of biomass components, which limit their green and high-value applications [4].

Deep eutectic solvents (DESs) have emerged as a promising class of green and designable solvents for lignocellulosic biorefineries [5]. In general, DESs are formed through intermolecular interactions between hydrogen-bond acceptors and hydrogen-bond donors, and they usually exhibit low volatility, tunable physicochemical properties, facile preparation, and relatively low cost [6]. Compared with conventional volatile organic solvents and, in some cases, ionic liquids, DES systems have shown considerable potential for selectively disrupting interactions within plant cell walls and promoting the fractionation of cellulose, hemicellulose, and lignin under relatively mild conditions [7]. In recent years, DES-based pretreatment and fractionation strategies have been increasingly explored for biomass conversion. Properly designed DESs can facilitate cellulose enrichment, promote hemicellulose and lignin removal, and improve the downstream conversion efficiency of lignocellulosic feedstocks [8].

Among different DES systems, alkaline DESs can promote the cleavage of ether and ester bonds in lignin. Among amine-based DESs, MEA-based DESs exhibit relatively high pH values and are efficient for lignin removal [9]. Sun and co-workers comparatively studied the pretreatment efficiency of acidic and basic DESs and indicated that basic DESs showed excellent performance for wheat straw pretreatment [10]. Microwave-assisted ChCl-MEA pretreatment of industrial xylose residue enabled tailored valorization, achieving 94% delignification, 90% cellulose saccharification, and lignin nanoparticles with excellent antioxidant activity [11]. ChCl-MEA pretreatment of poplar increased cellulose enzymatic digestibility 6.6-fold and extracted hemicelluloses with substituents [12]. However, the delignification efficiency of ChCl-MEA toward bamboo is relatively lower than that reported for some other biomass feedstocks [13]. This suggests that the ChCl-MEA system still requires further regulation to improve its applicability to bamboo pretreatment.

Introducing functional third components into the ChCl-MEA system may provide a practical way to tune solvent alkalinity, polarity, hydrogen-bonding ability, and lignin-interaction behavior. As reaction media for lignocellulosic biomass fractionation, solubilization performance and lignin structural integrity are critical factors in DES design. Previous studies indicate that increasing the basicity of DESs could promote the cleavage of ester bonds in lignin and glycosidic bonds in carbohydrates, thereby improving delignification. Thus, Na_2_S, as an alkaline enhancer and a strong reducing nucleophile, has been broadly used in the pulping industry because of its superior delignification ability and high carbohydrate preservation [14]. Considering the difficulty in treating Na_2_S-containing black liquor, NaHCO_3_-assisted DES pretreatment may provide a milder alternative strategy [15]. Hydrogen peroxide, which is widely used in biomass pretreatment, can enhance DES polarity, improve the ability of DESs to establish nonspecific interactions with biomass components, and introduce oxidative modification of biomass components [16]. Moreover, various studies suggest that ethylene glycol shows good lignin-dissolving ability and helps preserve *β*-O-4 linkages in lignin [17,18].

Apart from the excellent lignin solubility of alkaline DESs, such solvents also exhibit great potential for depolymerizing lignocellulosic components [13]. The degradation products may contain aromatic and hydroxyl groups, which can competitively form hydrogen bonds with DESs and thereby affect pretreatment efficiency. At the same time, the recovered DES-containing liquor also contains dissolved biomass-derived fragments and solvent components, which may be further valorized instead of being regarded only as waste liquor. Accordingly, both the degradation products and DESs can serve as carbon precursors, enabling the development of a waste-free biorefinery system [19].

Therefore, in this study, H_2_O_2_, NaHCO_3_, Na_2_S, and ethylene glycol (EG) were introduced into a choline chloride–monoethanolamine (ChCl-MEA) DES system for pretreatment of bamboo. The effects of different third components on the physicochemical properties of ChCl-MEA-based solvent systems were investigated. The efficiency of the third-component-regulated ChCl-MEA pretreatment was assessed based on biomass fractionation, cellulose accessibility, enzymatic saccharification, ethanol fermentation, and recovered-liquor valorization. This work aims to establish a clearer link between third-component regulation, substrate structural reconstruction, and bioethanol-oriented bamboo conversion, while also exploring carbon-dot preparation as an auxiliary pathway for recovered-liquor valorization.

## 2. Results and Discussion

### 2.1. Effect of Pretreatment on Structural Characteristics of Bamboo

#### 2.1.1. Chemical Composition

**Figure 1:**
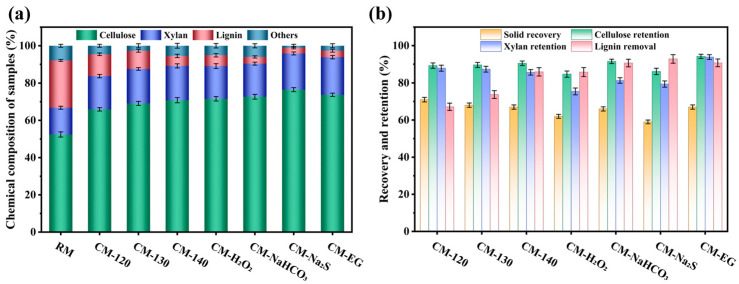
Changes in the chemical composition of bamboo residues after DES pretreatment. (**a**) Solid recovery, cellulose, xylan, and lignin contents of residues obtained from binary ChCl-MEA pretreatment at 120, 130, and 140 °C. (**b**) Comparison of residue composition and delignification performance for third-component-modified ChCl-MEA systems at 140 °C.

Deep eutectic solvents are robust solvents for biomass fractionation, prioritizing delignification while preserving cellulose. As depicted in Figure 1, the chemical composition of bamboo and its derived residues undergoes dramatic changes upon pretreatment. Raw bamboo contains 52.4 ± 0.6% cellulose, 14.4 ± 0.4% xylans, and 25.3 ± 0.6% lignin. Treatment with ChCl-MEA resulted in significant delignification (from 67.1 ± 2.0% to 85.9 ± 2.3%) and cellulose enrichment (from 52.4 ± 1.5% to 70.8 ± 1.2%) as temperatures rose from 120 °C to 140 °C, albeit with a marginal decline in solid recovery (71.0 ± 1.6% to 67.0 ± 1.5%). The outstanding delignification efficiency is likely due to the facilitated solvent penetration into the cell wall at higher temperatures, which concomitantly accelerates the degradation of ether linkages within lignin and lignin–carbohydrate complexes (LCCs) [20,21]. However, 85.6 ± 1.6% to 87.8 ± 1.8% xylan retention was achieved during pretreatment, demonstrating the efficacy of this alkaline DES system in hemicellulose preservation [22].

Compared with the binary system, the introduction of a third component further improves the fractionation performance of ChCl-MEA and promotes lignocellulose deconstruction effectively at 140 °C. Among the ChCl-MEA-based solvent systems containing different third components, ChCl-MEA-Na_2_S achieves the highest delignification rate (92.8 ± 2.3%), while ChCl-MEA-EG (90.7 ± 2.1%) and ChCl-MEA-NaHCO_3_ (90.6 ± 2.1%) also show favorable lignin removal efficiencies, all outperforming ChCl-MEA-H_2_O_2_ (85.8 ± 2.4%). The superior performance of the Na_2_S-containing system can be attributed to the strong nucleophilicity and mild reducing character of sulfide species (HS^−^ and S2^−^), which can promote β-O-4 ether bond cleavage through thiolysis-like reactions while suppressing lignin condensation and redeposition. The addition of Na_2_S therefore provides ChCl-MEA with a stronger nucleophilic, reductive, and alkaline microenvironment, which is favorable for delignification [23]. By contrast, the H_2_O_2_ system relies mainly on radical oxidation and may cause partial polysaccharide degradation; NaHCO_3_ provides moderate basicity and has a limited effect on ether bond cleavage; and EG mainly acts as a co-solvent to regulate viscosity, with relatively weak chemical reactivity [24]. Overall, ChCl-MEA-Na_2_S exhibits the most pronounced pretreatment effect at 140 °C, giving a solid recovery of 59.0 ± 1.7%, with the recovered solid containing 76.5 ± 1.6% cellulose, 19.4 ± 1.2% xylan, and 3.1 ± 0.5% lignin.

**Figure 2:**
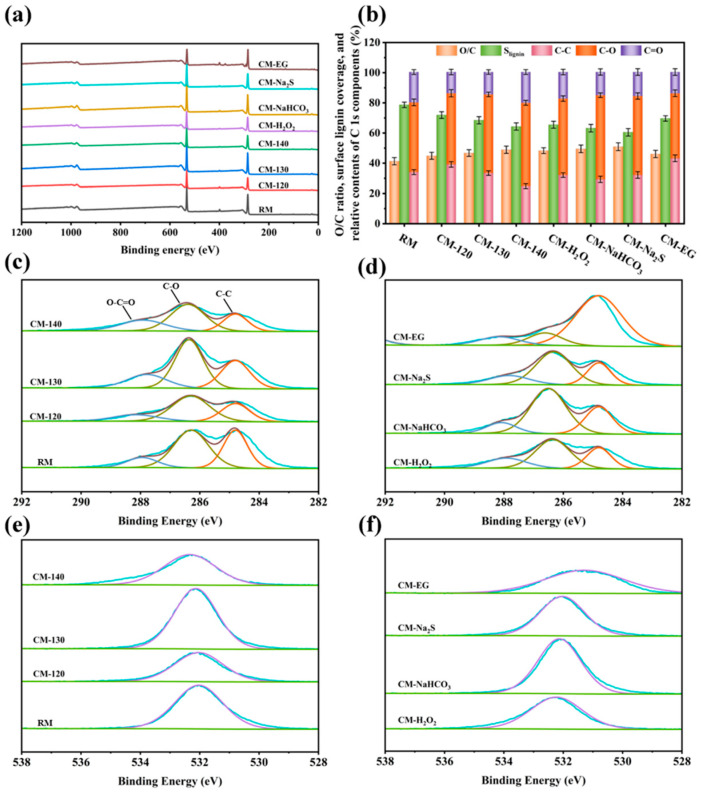
XPS characterization of raw bamboo and DES-pretreated residues. (**a**) XPS survey spectra of raw and pretreated samples. (**b**) XPS-derived O/C ratio, surface lignin coverage, and relative contents of C 1s components. (**c**,**d**) High-resolution C 1s spectra of residues obtained from the binary and third-component-modified ChCl-MEA systems, respectively; the colored fitting curves represent the deconvoluted C–C, C–O, and O–C=O components. (**e**,**f**) High-resolution O 1s spectra of residues obtained from the binary and third-component-modified ChCl-MEA systems, respectively.

The changes in chemical composition are also confirmed by FT-IR and XPS (Figure 2 and Figure S1). The intensity of the peak at 1730 cm^−1^ decreases after pretreatment, indicating removal of hemicellulose. Meanwhile, the decline in the intensity of the peak at 1519 cm^−1^ confirmed delignification of DES pretreatment [25] (Figure S1). Consistent with the chemical composition, delignification leads to a decrease in content of C–C/C–H groups from 34.0 ± 2.1% to 24.6 ± 2.1% and an increase in O/C ratio from 0.413 ± 0.024 to 0.490 ± 0.025 as the pretreatment temperature increases from 120 to 140 °C. The surface lignin coverage also decreases from 78.7 ± 2.1% to 63.8 ± 2.5% as the pretreatment temperature increases. These changes indicate that increasing pretreatment severity progressively reduced lignin shielding at the residue surface and promoted the exposure of oxygen-containing polysaccharide-related functionalities [26]. In coordination with the third component, the O/C ratio further increases but differs with the properties of the third component. CM-H_2_O_2_ and CM-NaHCO_3_ exhibit O/C ratios of 0.489 ± 0.019 and 0.500 ± 0.026, together with surface lignin coverages of 65.2 ± 2.3% and 62.4 ± 2.5%, respectively, indicating more oxygenated and less lignin-covered surfaces than those of the corresponding binary residues. CM-Na_2_S has the highest O/C ratio (0.507 ± 0.026) and the lowest surface lignin coverage (59.6 ± 2.5%), confirming that this system most effectively reduces surface lignin shielding. In contrast, CM-EG shows an O/C ratio of 0.466 ± 0.022, while its surface lignin coverage remains at 68.9 ± 2.4% and the C–C/C–H contribution rose to 42.8 ± 2.2%. This discrepancy between bulk delignification and surface composition suggests that dissolved lignin fragments are partially re-deposited on the outer surface during post-treatment [27,28]. Among the third-component-modified ChCl-MEA systems, the Na_2_S-containing DES generates the most favorable surface characteristics for reducing lignin shielding, whereas the EG-containing system retains a more lignin-enriched outer layer that could hinder enzyme accessibility.

#### 2.1.2. Changes in Degree of Polymerization and Crystalline Structure

**Figure 3:**
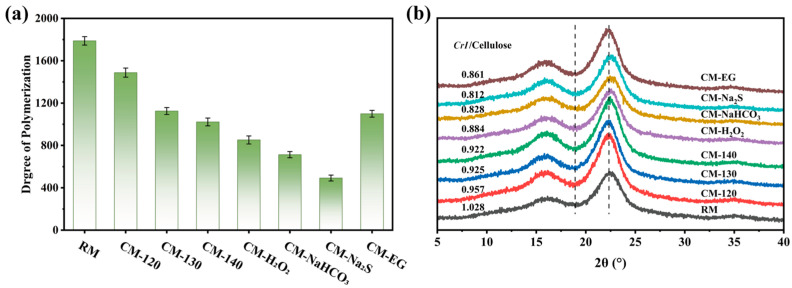
Changes in cellulose degree of polymerization (**a**) and crystalline structure of bamboo residues (**b**) after DES pretreatment.

DES pretreatment not only alters the chemical composition of bamboo residues but also affects the structure of cellulose. Figure 3 depicts the effect of DES pretreatments on the degree of polymerization (DP) and crystalline index of samples. The DP of raw bamboo is 1788 ± 40, which decreases to 1488 ± 43 (CM-120), 1125 ± 33 (CM-130), and 1022 ± 37 (CM-140) after binary ChCl-MEA pretreatment, indicating that cellulose chains are progressively cleaved as the pretreatment severity increased. In the third-component-modified ChCl-MEA systems, this trend becomes more pronounced: the DP further decreases to 852 ± 38 for CM-H_2_O_2_, 713 ± 29 for CM-NaHCO_3_, and 492 ± 28 for CM-Na_2_S, whereas CM-EG still retains a relatively higher value of 1099 ± 32. The decrease in DP indicates that DES treatment weakened the constraints between cellulose chains, making the chain segments more relaxed and exposed, while also further opening the originally compact internal structure of the substrate. Among all the systems, CM-Na_2_S shows the lowest DP, suggesting that this ChCl-MEA-Na_2_S caused the most extensive disruption of the bonding structures within the bamboo cell wall [29], which is consistent with its higher delignification efficiency and stronger enzymatic hydrolysis performance.

XRD spectra reflect the changes in the ordered structure of cellulose. All samples retain the characteristic diffraction peaks of cellulose I at around 18.8° and 22.5°, indicating that DES pretreatments do not alter the cellulose crystal form. Since pretreatment changed the cellulose content of the residues, the apparent *CrI* value could be affected by cellulose enrichment caused by the removal of hemicellulose and lignin. Therefore, *CrI*/cellulose content was used in this study as a normalized crystallinity-related parameter to compare the relative crystallinity variation among samples with different cellulose contents. The raw bamboo exhibits a *CrI*/Cellulose value of 1.028, which decreases to 0.957 (CM-120), 0.925 (CM-130), and 0.922 (CM-140) after binary ChCl-MEA pretreatment, suggesting that the internal ordered hydrogen-bonding network is partly disturbed. In the third-component-modified ChCl-MEA systems, this value further decreases to 0.884 (CM-H_2_O_2_), 0.861 (CM-EG), 0.828 (CM-NaHCO_3_), and 0.812 (CM-Na_2_S). The more pronounced decreases observed for the NaHCO_3_ and Na_2_S systems indicate a stronger disruption of the highly ordered cellulose regions, which is more favorable for chain swelling and substrate opening [30]. Taken together with the DP results, DES pretreatments promote cellulose chain scission also weaken the ordered packing within its supramolecular structure, and this effect was most pronounced in the ChCl-MEA-Na_2_S system.

#### 2.1.3. Specific Surface Area, Hydrophobicity, and Enzyme–Substrate Interactions

**Figure 4:**
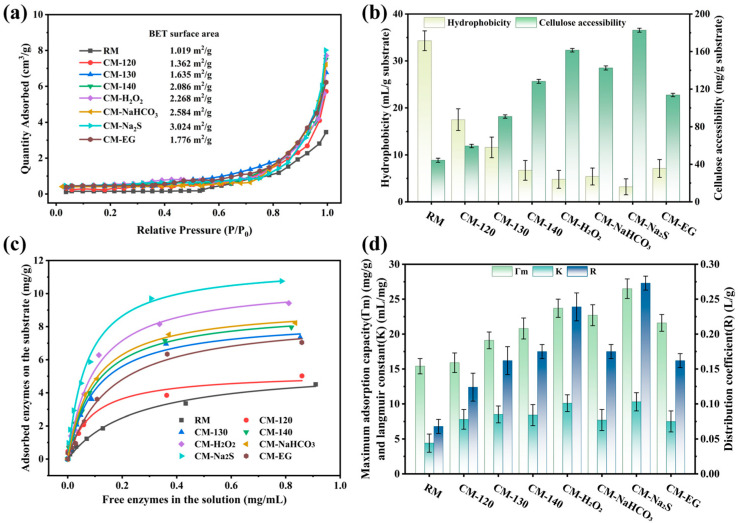
Changes in the interfacial and enzymatic properties of bamboo residues after DES pretreatment. (**a**) N_2_ adsorption isotherms and BET specific surface areas. (**b**) Surface hydrophobicity and cellulose accessibility of raw and pretreated residues. (**c**) Langmuir adsorption isotherms of cellulase on raw and pretreated residues. (**d**) Langmuir adsorption behavior of cellulase on raw and pretreated residues.

The changes in the intrinsic structure of cellulose also affect interfacial properties of samples. After pretreatment, the N_2_ adsorption isotherms and the corresponding BET specific surface areas, as well as the hydrophobicity, cellulose accessibility, and enzyme adsorption behavior of the samples, are presented in Figure 4. BET analysis shows that pretreatment with ChCl-MEA increases the specific surface area of samples from 1.019 m^2^/g to 1.362 m^2^/g (CM-120), 1.635 m^2^/g (CM-130), and 2.086 m^2^/g (CM-140), respectively, indicating that the cell-wall structure is gradually loosened and generates accessible pores as the pretreatment severity increased. In the third-component-modified ChCl-MEA systems, the specific surface area further increases to 2.268 m^2^/g (CM-H_2_O_2_), 2.584 m^2^/g (CM-NaHCO_3_), 3.024 m^2^/g (CM-Na_2_S), and 1.776 m^2^/g (CM-EG), respectively.

Correspondingly, the cellulose accessibility increases markedly from 44.3 ± 2.2 mg/g for raw bamboo to 59.5 ± 1.8 mg/g (CM-120), 90.8 ± 1.8 mg/g (CM-130), and 128.2 ± 2.1 mg/g (CM-140), respectively. In the third-component-modified ChCl-MEA systems, it further increases to 161.4 ± 1.9 mg/g (CM-H_2_O_2_), 142.5 ± 2.2 mg/g (CM-NaHCO_3_), and 182.7 ± 2.1 mg/g (CM-Na_2_S), while CM-EG shows 113.7 ± 1.8 mg/g. The increase in cellulose accessibility is much greater than the increase in specific surface area, indicating that enzyme access to the substrate was governed not only by pore enlargement but also by cellulose chain relaxation, reduced lignin shielding, and improved interfacial chemical properties.

The hydrophobicity results further reveal the changes in the surface properties of the residues. The hydrophobicity of raw bamboo is 34.3 ± 2.1 L/g, which decreases to 17.5 ± 2.3 L/g (CM-120), 11.6 ± 2.2 L/g (CM-130), and 6.7 ± 2.1 L/g (CM-140) after binary ChCl-MEA pretreatment. In the third-component-modified ChCl-MEA systems, it further decreases to 4.8 ± 1.9 L/g (CM-H_2_O_2_), 5.4 ± 1.8 L/g (CM-NaHCO_3_), and 3.2 ± 1.7 L/g (CM-Na_2_S), whereas CM-EG shows 7.1 ± 1.9 L/g. The decrease in hydrophobicity indicates that the lignin-rich hydrophobic domains on the substrate surface are reduced, while polar groups became more exposed, thereby improving interfacial wettability. This change could not only facilitate enzyme access to the substrate but also weaken the non-productive adsorption caused by residual lignin [31].

Structural and interfacial changes reflect the enzyme adsorption behavior. For raw bamboo, the maximum adsorption capacity (*Γ*m), affinity constant (K), and R value are 15.4 ± 1.1 mg/g, 4.4 ± 1.3 mL/mg, and 0.068 ± 0.01 L/g, respectively. After binary ChCl-MEA pretreatment, *Γ*m increases to 15.9 ± 1.4, 19.1 ± 1.2, and 20.8 ± 1.5 mg/g; K increases to 7.8 ± 1.4, 8.5 ± 1.2, and 8.4 ± 1.5 mL/mg; and R increases to 0.124 ± 0.02, 0.162 ± 0.02, and 0.175 ± 0.01 L/g for CM-120, CM-130, and CM-140, respectively. In the third-component-modified ChCl-MEA systems, *Γ*m reaches 23.7 ± 1.3, 22.7 ± 1.5, and 26.5 ± 1.4 mg/g for CM-H_2_O_2_, CM-NaHCO_3_, and CM-Na_2_S, with corresponding K values of 10.1 ± 1.2, 7.7 ± 1.5, and 10.3 ± 1.3 mL/mg and R values of 0.239 ± 0.02, 0.175 ± 0.01, and 0.273 ± 0.01 L/g, respectively; CM-EG shows 21.6 ± 1.2 mg/g, 7.5 ± 1.5 mL/mg, and 0.162 ± 0.01 L/g.

Overall, the ChCl-MEA-Na_2_S pretreatment exhibits the highest effect on specific surface area, cellulose accessibility and enzyme adsorption capacity and affinity among all DES pretreatments. The pore opening, surface hydrophilization, and exposure of reactive sites boost the enzyme–substrate interactions, which will enhance the saccharification efficiency of cellulose [32].

### 2.2. Effect of Pretreatment on Cellulose Saccharification and Bioethanol Production

**Figure 5:**
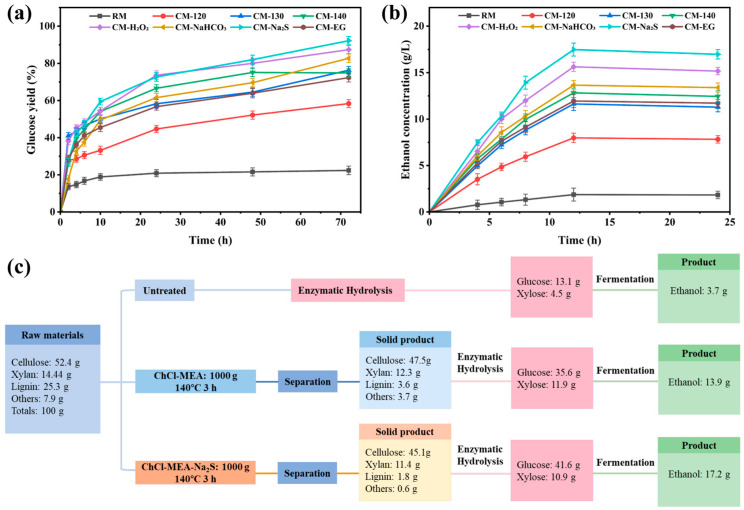
Enzymatic saccharification and ethanol fermentation performance of raw and DES-pretreated bamboo. (**a**) Glucose yields after enzymatic hydrolysis. (**b**) Ethanol concentration after fermentation. (**c**) Mass balance of bamboo conversion after DES pretreatment, enzymatic hydrolysis, and ethanol fermentation.

The effect of pretreatments on enzymatic saccharification of cellulose and bioethanol production is depicted in Figure 5, and the corresponding xylose yields are shown in Figure S2. Without pretreatment, the glucose and xylose yields of RM via enzymatic hydrolysis are only 22.4 ± 2.3% and 27.6 ± 1.8%, respectively, indicating that the dense lignin–hemicellulose matrix in native bamboo severely limits the action of enzymes on the polysaccharide fraction. After pretreatment with the binary ChCl-MEA, the glucose yield increases to 58.3 ± 2.1% (CM-120), 76.3 ± 2.1% (CM-130), and 74.9 ± 2.2% (CM-140), while the xylose yield increases to 84.9 ± 1.8% (CM-120), 97.2 ± 1.9% (CM-130) and 97.3 ± 1.9% (CM-140). These results suggest that increasing the pretreatment severity can markedly improve the enzymatic saccharification efficiency of carbohydrate fractions. Pretreatment with third-component-modified ChCl-MEA systems further enhanced cellulose conversion. The yields of glucose from CM-H_2_O_2_, CM-EG, CM-NaHCO_3_, and CM-Na_2_S via enzymatic hydrolysis are 87.4 ± 2.1%, 72.3 ± 2.3%, 82.8 ± 2.3%, and 92.2 ± 2.2%, respectively, while the corresponding ethanol concentrations were 15.63 ± 0.5, 11.95 ± 0.7, 13.66 ± 0.5, and 17.49 ± 0.7 g/L. The high bioconversion of samples obtained from pretreatment with third-component-modified ChCl-MEA systems is ascribed to the enhancement in specific surface area, cellulose accessibility and enzyme adsorption capacity and affinity of substrates. Among these samples, the substrate pretreated with ChCl-MEA-Na_2_S DES at 140 °C possesses excellent bioconversion capability.

Taking 100 g of raw bamboo as the basis, the mass balance of enzymatic hydrolysis and fermentation is summarized in Figure 5c. Direct enzymatic hydrolysis of untreated bamboo released only 13.1 g of glucose and 4.5 g of xylose, and subsequent fermentation produced 3.7 g of ethanol. After pretreatment with ChCl-MEA at 140 °C for 3 h, the obtained cellulose-rich residue contained 47.5 g of cellulose, 12.3 g of xylan, 3.6 g of lignin, and 3.7 g of other components. Enzymatic hydrolysis of this residue released 35.6 g of glucose and 11.9 g of xylose, and subsequent fermentation produced 13.9 g of ethanol. The incorporation of Na_2_S into the ChCl-MEA system further improved substrate digestibility. The ChCl-MEA-Na_2_S-treated residue contained 45.1 g of cellulose, 11.4 g of xylan, 1.8 g of lignin, and 0.6 g of other components, and its enzymatic hydrolysis released 41.6 g of glucose and 10.9 g of xylose. After fermentation, the ethanol output increased to 17.2 g based on 100 g of raw bamboo. These results indicate that Na_2_S-assisted ChCl-MEA pretreatment promoted lignin removal and improved cellulose accessibility, thereby enhancing the overall conversion of bamboo biomass into fermentable sugars and ethanol.

### 2.3. Molecular Interpretation of Third-Component-Modified ChCl-MEA System Performance

**Figure 6:**
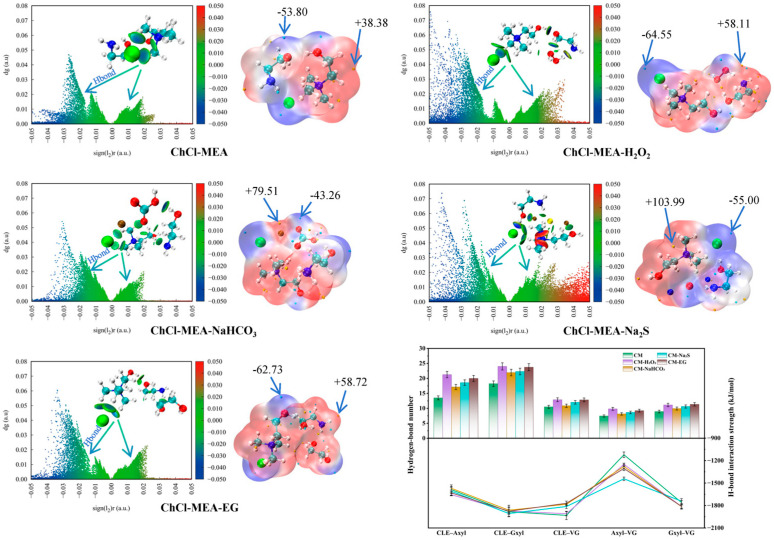
RDG isosurfaces, molecular electrostatic potential (MEP) distributions, and hydrogen-bond number and cumulative interaction energy analyses of ChCl-MEA and third-component-modified ChCl-MEA systems.

To further understand the interactions between DESs and lignocellulosic substrate, reduced density gradient (RDG)-based noncovalent interaction analysis, molecular electrostatic potential (MEP), hydrogen-bond statistics, Kamlet–Taft parameters, and NMR were performed. The RDG, MEP, hydrogen-bond, and interaction-energy results are shown in Figure 6, the Kamlet–Taft parameters, pH, and viscosity are shown in Figure S3, and the NMR results are shown in Figures S4 and S5. The Kamlet–Taft parameters of ChCl-MEA are α = 1.05 ± 0.04, *β* = 0.51 ± 0.03, π* = 0.93 ± 0.04, respectively. The pH and viscosity of ChCl-MEA are 11.7 ± 0.2 and 4.8 ± 0.3 mPa·s. Coordination with Na_2_S increases the acidity, basicity and polarity (α = 1.12 ± 0.06, *β* = 0.54 ± 0.02, π* = 1.42 ± 0.04, pH = 12.3 ± 0.3), indicating a more polarized and strongly basic environment. ChCl-MEA-NaHCO_3_ shows relatively high polarity with π* = 1.35 ± 0.06 and pH = 12.4 ± 0.03, while ChCl-MEA-H_2_O_2_ exhibited the highest α value of 1.58 ± 0.08, suggesting stronger hydrogen-bond donor ability.

As shown in Figure 6, all DESs exhibit obvious attractive interaction regions, confirming that hydrogen bonding and electrostatic interactions are the major forces stabilizing the eutectic systems. The H_2_O_2_-containing system shows a relatively uniform enhancement of attractive regions, suggesting an overall increase in polarity, whereas the NaHCO_3_-containing system displays more localized asymmetry. The Na_2_S-containing DES exhibits the most concentrated and strongest attractive regions, indicating the formation of a more strongly polarized local microenvironment. By contrast, the EG-containing DES shows a limited enhancement, implying that its role is more closely related to hydrogen-bond reorganization than to strong chemical activation. The MEP results further support these differences. For binary ChCl-MEA, the minimum and maximum electrostatic potentials are −53.80 and +38.38 kcal/mol, respectively. After introducing H_2_O_2_, the minimum negative potential deepens to −64.55 kcal/mol and the maximum positive potential increases to +58.11 kcal/mol, indicating a stronger contrast between electron-rich and electron-deficient regions and thus a greater tendency to interact with polar carbohydrate groups. In the NaHCO_3_ system, the minimum negative potential is −43.26 kcal/mol, whereas the maximum positive potential rises to +79.51 kcal/mol, reflecting a more asymmetric local electric field. The Na_2_S system shows the highest maximum positive potential (+103.99 kcal/mol), while the minimum negative potential remains at about −55.00 kcal/mol, indicating that it could generate the strongest potential gradient around aryl-ether bonds and is favorable for activating and cleaving lignin-related linkages. In contrast, the EG system shows a weaker enhancement in polarity, suggesting that EG contributes mainly to viscosity reduction, swelling promotion, and hydrogen-bond reorganization rather than direct bond cleavage [33]. The hydrogen-bond and cumulative interaction-energy statistics in Figure 6 are also consistent with these results: H_2_O_2_ and EG show stronger interactions in the carbohydrate-related models, whereas Na_2_S exhibits the highest cumulative interaction energy in the Axyl-VG model, indicating a stronger directed interaction with lignin aromatic ether structures.

This interpretation is also supported by the NMR results. As shown in Figure S4, the ^1^H and ^13^C NMR spectra of the recovered DESs largely retain the main characteristic signals of the fresh systems, although weak additional signals and peak-intensity variations can be observed in some recovered samples, indicating that the main DES framework is generally preserved after pretreatment. The broadening and intensity variations of peaks around 3–4 ppm suggest rearrangement of the local hydrogen-bonding environment. In addition, the intensity of cross-peaks in the δ 2.5–4.0 ppm region decreases after pretreatment, suggesting a decrease in hydrogen-bond intensity and reconstruction of hydrogen bonds [34]. Further evidence is provided by the 2D NOESY spectra (Figure S5). Compared with the fresh DESs, the recovered systems still retain identifiable intermolecular cross-peaks, although the cross-peak intensity becomes weaker and more dispersed after pretreatment, indicating that the DES framework is preserved while the spatial proximity and hydrogen-bonding network are reorganized to different extents. In all, the excellent pretreatment performance of the DESs ChCl-MEA-Na_2_S and ChCl-MEA-H_2_O_2_ is due to the enhancement of polarity, hydrogen-bond capacity.

### 2.4. Structure and Properties of Carbon Dots (CDs) Prepared from Recovered DESs

**Figure 7:**
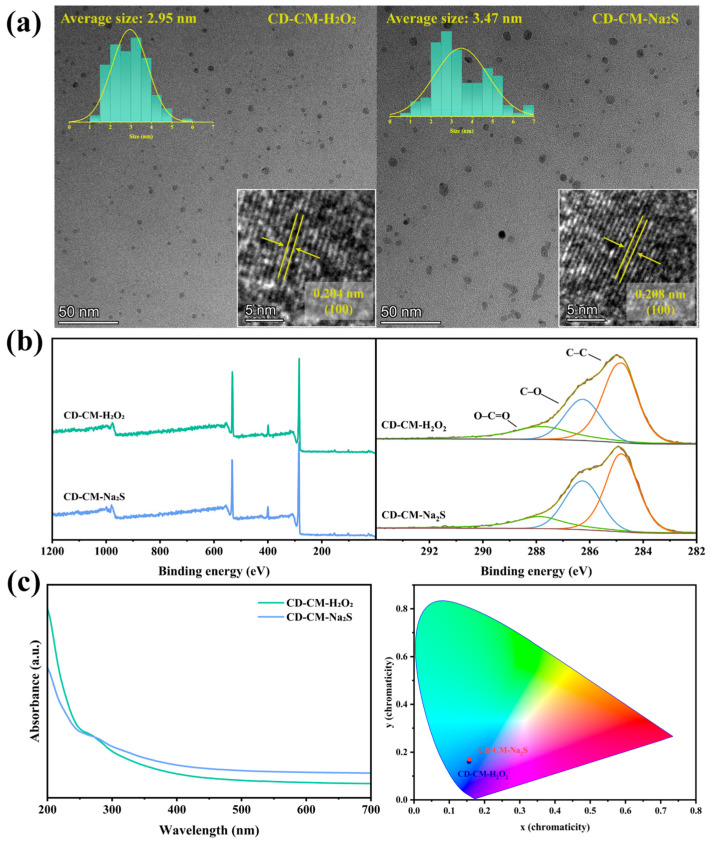
(**a**) TEM and HRTEM images of CDs. (**b**) XPS and related surface-chemical characterization of carbon dots; the colored fitting curves indicate different C 1s components. (**c**) UV–Vis absorption and fluorescence emission behaviors of carbon dots.

Due to the high fractionation performance of DES ChCl-MEA-H_2_O_2_ and ChCl-MEA-Na_2_S systems, the degraded components of bamboo in these DESs are converted into carbon dots for full utilization of lignocellulosic biomass. Both CDs exhibit well-dispersed quasi-spherical morphologies at the nanoscale. The average particle size of the carbon dots derived from the ChCl-MEA-H_2_O_2_ and ChCl-MEA-Na_2_S system are 2.95 nm and 3.47 nm, respectively (Figure 7a). The lattice spacings of the CD-CM-H_2_O_2_ and CD-CM-Na_2_S are 0.204 and 0.208 nm, close to the spacing of graphitic carbon (100), suggesting that both samples possessed a certain degree of ordered carbon structure. The slightly larger lattice spacing in the Na_2_S-derived carbon dots may be due to the condensation of lignin in the CD-CM-Na_2_S DES, which leads to defects or local structural disorder [35].

The functional groups on the surface of CDs are detected via XPS analysis and shown in Figure 7b. C and O are the main elements in CDs, and C-C/C=C, C-O, and O-C=O are the main bonds on the CD surface. Compared with the CD-CM-Na_2_S, a higher proportion of oxygen-containing species is observed in CD-CM-H_2_O_2_, indicating a stronger degree of surface oxidation [36].

In terms of optical properties (Figure 7c), both samples show obvious absorption peaks in the range of 200–300 nm, corresponding to π-π* transitions in the carbon framework, while the absorption tails at longer wavelengths are associated with surface defect states and n-π* transitions. Fluorescence measurements show that both carbon dots exhibited emission peaks near 450 nm, but the sample derived from the H_2_O_2_ system shows higher fluorescence intensity, indicating that its surface-state distribution and defect structure are more favorable for fluorescence emission.

**Figure 8:**
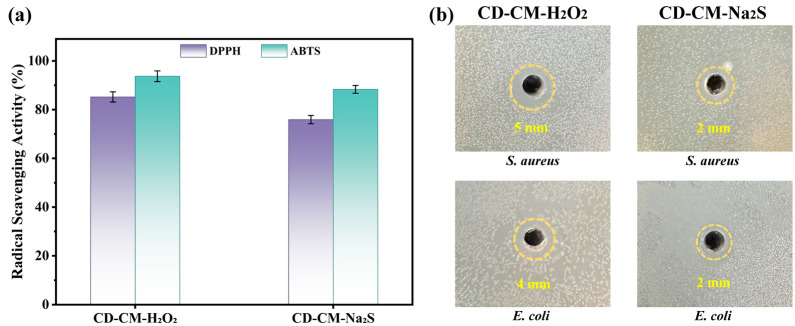
Bioactivities of carbon dots. (**a**) Radical-scavenging activities evaluated by DPPH and ABTS assays. (**b**) Antibacterial activity against *S. aureus* and *E. coli*.

As shown in Figure 8, the functional groups on the surface also contribute to the bioactivity of CDs. The DPPH and ABTS radical-scavenging rates of the H_2_O_2_-derived carbon dots reach 85.2 ± 2.1% and 93.7 ± 2.2%, respectively, which were higher than those of the Na_2_S-derived sample (75.9 ± 1.7% and 88.3 ± 1.6%). This indicates that the former possessed stronger antioxidant activity, which may be related to its smaller particle size and higher content of surface oxygen-containing functional groups [37,38]. In the antibacterial tests, the Na_2_S-derived carbon dots produce inhibition zones of 2 mm against both *S. aureus* and *E. coli*, whereas the H_2_O_2_-derived sample produced inhibition zones of 5 and 4 mm, respectively, indicating stronger antibacterial activity. These results suggest that the degradation component from lignocellulosic biomass during DES pretreatments can be used as carbon sources for carbon dot preparation. The physicochemical properties of CDs are also related to the properties of DESs. In all, the recovered ChCl-MEA-H_2_O_2_ DES after pretreatment is favorable for bioactive CD production due to the oxidation of H_2_O_2_.

## 3. Materials and Methods

### 3.1. Materials

*Dendrocalamus brandisii* was collected from the bamboo garden of Southwest Forestry University (Kunming, China). The raw material was naturally air-dried, pulverized, and sieved to 40–60 mesh prior to use. Choline chloride (ChCl, ≥98%), monoethanolamine (MEA, ≥99.5%), hydrogen peroxide solution (H_2_O_2_, 30 wt%), sodium bicarbonate (NaHCO_3_, ≥99.5%), sodium sulfide nonahydrate (Na_2_S·9H_2_O, ≥98%), and ethylene glycol (EG, ≥99.5%) were purchased from Sinopharm Group Co., Ltd. (Shanghai, China). Commercial cellulase preparations Cellic CTec2 (200 FPU/mL) and Celluclast 1.5 L (40 mg/mL) were obtained from Sigma-Aldrich (Shanghai, China). *Saccharomyces cerevisiae* used for ethanol fermentation was purchased from Angel Yeast Co., Ltd. (Yichang, China). *Escherichia coli* (*E. coli*) and *Staphylococcus aureus* (*S. aureus*) strains used for antibacterial assays were obtained from Beijing Baochang Biotechnology Co., Ltd. (Beijing, China).

### 3.2. Preparation and Characterization of DESs

The ChCl-MEA (CM) deep eutectic solvent was prepared by mixing choline chloride and monoethanolamine at a molar ratio of 1:6. For the ChCl-MEA-based solvent systems containing different third components, H_2_O_2_, NaHCO_3_, Na_2_S and EG were individually added to the ChCl-MEA DES system as the third component, with a molar ratio of 0.2 relative to the ChCl. Then, the mixtures were magnetically stirred in a water bath at 80 °C until a transparent, homogeneous liquid was formed.

The Kamlet–Taft parameters (*α*, *β*, and π*) of the DESs were determined using the solvatochromic probes 4-nitroaniline, N,N-diethyl-4-nitroaniline, and Nile Red according to Teles et al. [39]. To provide a molecular-level interpretation of the interactions between DESs and lignocellulosic components, representative computational analyses were performed. The computational models were simplified molecular models constructed to qualitatively compare local interaction tendencies rather than to reproduce the complete composition of the experimental solvent formulations. The molecular structures were geometrically optimized using ORCA 4.0 software at the B3LYP-D3(BJ)/def2-SVP level of theory [40]. Electrostatic potential (ESP), reduced density gradient (RDG), hydrogen-bonding tendency, and interaction-energy analyses were conducted to compare the interaction characteristics of different DES systems with lignocellulosic model compounds. The ESP and RDG results were analyzed using Multiwfn ver. 3.8 [41], and the noncovalent interaction regions were visualized using VMD ver. 1.9.3 software [42]. Veratryl glycerol-*β*-guaiacyl ether (VG), 4-O-methylglucurono-xylan, and cellobiose were selected as representative model compounds for lignin, hemicellulose, and cellulose, respectively [43]. Therefore, these computational results were used only as supporting evidence for interpreting the relative interaction tendencies among different solvent systems.

### 3.3. Pretreatments

For the pretreatment step, the pretreatment temperature was first optimized. Briefly, 10 g of *D. brandisii* powder was mixed with 100 g of ChCl-MEA, and the mixture was incubated in an oil bath at 120 °C, 130 °C, and 140 °C for 3 h, respectively. Pretreatments using the third-component-modified ChCl-MEA systems were performed at 140 °C for 3 h. At the end of the reaction, the solid residue was collected by filtration and successively washed with 300 mL of acetone/water (1:1, *v*/*v*) and hot deionized water until the filtrate approached neutral pH. The resulting cellulose-rich solids were dried and labeled according to the temperature or the third component as CM-X (X representing the adopted pretreatments or third components). The untreated raw bamboo powder was taken as the control and labeled as RM. The filtrate was adjusted to pH 2 with hydrochloric acid to precipitate lignin, which was then collected by centrifugation. The supernatant was concentrated by rotary evaporation to remove water and recover the DESs for carbon dot (CD) production.

For CD preparation, the recovered ChCl-MEA-H_2_O_2_ and ChCl-MEA-Na_2_S were diluted to a 10 wt% aqueous solution, transferred into a Teflon-lined stainless-steel autoclave, and heated at 180 °C for 12 h. After cooling to room temperature, the obtained dispersion was centrifuged, filtered through a 0.22 μm membrane, and dialyzed against deionized water before further characterization. The obtained CDs were labeled as CD-CM-H_2_O_2_ and CD-CM-Na_2_S, respectively.

### 3.4. Enzymatic Hydrolysis and Fermentation

Enzymatic hydrolysis was performed in sodium citrate buffer (pH 4.8) at a solids loading of 5% (*w*/*v*) according to a previously reported method with slight modifications [44]. Cellic CTec2 was dosed at 15 FPU/g substrate, and the reaction was conducted at 50 °C with shaking at 150 rpm for 72 h; hydrolysates were withdrawn periodically during the process. At the end of the enzymatic saccharification, the hydrolysates were filtered and sterilized at 121 °C prior to inoculation with *Saccharomyces cerevisiae*. The yeast suspension was prepared by dissolving 0.21 g of yeast, 0.40 g of glucose, and 0.80 g of yeast extract in 8 mL of deionized water, followed by activation at 35 °C for 30 min. Then, 4 mL of sterilized hydrolysate was inoculated with 0.5 mL of the activated yeast suspension before fermentation. Fermentation was carried out at 35 °C with shaking at 90 rpm for 24 h, and supernatant samples were collected at intervals for ethanol analysis. Glucose, xylose, and ethanol concentrations during enzymatic hydrolysis and fermentation were quantified using high-performance liquid chromatography (HPLC, Agilent 1260, Agilent Technologies, Santa Clara, CA, USA) equipped with a refractive index detector (RID) and a Bio-Rad Aminex HPX-87H column. A 5 mM H_2_SO_4_ aqueous solution was used as the mobile phase at a flow rate of 0.6 mL/min, and the column temperature was maintained at 55 °C [45]. Glucose, xylose, and ethanol were identified by comparing their retention times with those of authentic standards and quantified using external calibration curves. Glucose and xylose yields were calculated based on the carbohydrate contents of the corresponding substrates, and ethanol output in the mass balance was normalized to 100 g of raw bamboo.

### 3.5. Characterization

The composition of samples was determined in accordance with the NREL LAP protocol [45]. The hydrophobicity and enzyme accessibility of the cellulosic substrates were evaluated by Rose Bengal and Direct Red 28 staining, respectively [46,47]. The degree of polymerization (DP) of cellulose was calculated based on its intrinsic viscosity in a cupriethylenediamine (CED) solution [48]. The protein adsorption behavior of Celluclast 1.5 L (40 mg/mL protein content) on the samples was analyzed using the Langmuir adsorption isotherm model [49]. The maximum adsorption capacity (*Γ*m) and adsorption affinity constant (K) were obtained via Langmuir fitting, and the adsorption strength parameter R was calculated from *Γ*m and K.

The specific surface area of the raw and pretreated samples was measured by Brunauer–Emmett–Teller (BET) analysis using an ASAP 2460 surface area analyzer (Micromeritics Instrument Ltd., Norcross, GA, USA). The crystallinity index (CrI) of the raw and pretreated bamboo samples was determined by X-ray diffraction (XRD), and the relative change in crystallinity was expressed as the ratio of CrI to cellulose content (CrI/cellulose content). The surface elemental composition of the cellulosic substrates and CDs was analyzed by X-ray photoelectron spectroscopy (XPS, Thermo Scientific, Waltham, MA, USA) to determine the distribution of carbon and oxygen, as well as to calculate the surface coverage of lignin. Functional groups in the cellulosic substrates were characterized by Fourier transform infrared spectroscopy (FT-IR) over the wavenumber range of 4000–400 cm^−1^.

The morphology and microstructure of the CDs were characterized using a high-resolution transmission electron microscope (HRTEM, JEM-F200, JEOL Ltd., Tokyo, Japan) operated at 200 kV. Before observation, the purified CD dispersion with a predetermined concentration was dried and used for TEM/HRTEM analysis. The particle-size distribution was determined from TEM images using ImageJ ver. 1.54i software by randomly measuring 120 individual particles. The lattice spacing was estimated from the HRTEM image after calibration with the 5 nm scale bar by measuring ten consecutive lattice fringes.

The optical properties of CDs were investigated using a UV–Vis and a fluorescence spectrophotometer. The CDs were dispersed in deionized water until their optical density in the range of 300–400 nm was below 0.10, so as to minimize the inner filter effect. Subsequently, the absorption spectra of the CD dispersions in the range of 200–600 nm were recorded on a UV–Vis spectrophotometer at room temperature with an interval of 1 nm. The fluorescence spectra of CDs were measured using a fluorescence spectrophotometer. The excitation wavelength was scanned from 310 to 380 nm with a step size of 10 nm, and the emission spectra in the range of 400–600 nm were recorded at each excitation wavelength. Both the excitation and emission slit widths were set to 5 nm, the scanning rate was 200–300 nm/min, and the photomultiplier tube voltage was adjusted to avoid signal saturation. The excitation/emission pair corresponding to the maximum emission intensity was defined as the optimal excitation/emission wavelength.

Before antioxidant and antibacterial assays, the purified CD dispersions were adjusted to a concentration of 1.0 mg/mL. The antioxidant activity of the CDs was evaluated by 2,2-diphenyl-1-picrylhydrazyl (DPPH) and 2,2′-azinobis(3-ethylbenzothiazoline-6-sulfonic acid) (ABTS) radical-scavenging assays [50,51]. The antibacterial performance was assessed by the agar well diffusion method on Mueller–Hinton agar plates, using *S. aureus* and *E. coli* as the test strains [52]. Briefly, bacterial suspensions were adjusted to approximately 10^6^ CFU/mL and uniformly spread onto the agar plates. Wells with a diameter of 2 mm were punched into the agar, and 20 μL of CD dispersion was added to each well, followed by incubation at 37 °C for 24 h. Unless otherwise specified, quantitative experiments were performed in triplicate, and the results are reported as mean ± standard deviation where applicable. Detailed experimental procedures and calculation methods for the above assays are provided in the Supplementary Material.

## 4. Conclusions

This study demonstrates that the lignin dissolution and cellulose preservation during ChCl-MEA-based DES pretreatment can be effectively enhanced by introducing different third components. Multiscale characterizations and molecular simulation revealed that the enhanced performance stemmed from stronger disruption of lignin–carbohydrate complexes (LCCs), improved cellulose accessibility, reduced lignin shielding, and favorable enzyme–substrate interactions. Among the tested systems, ChCl-MEA-Na_2_S pretreatment at 140 °C for 3 h showed the best overall performance, affording 92.8 ± 2.3% delignification, 86.1 ± 1.7% cellulose recovery, 92.2 ± 2.2% cellulose saccharification, and an ethanol yield of 17.2 g per 100 g of raw bamboo. After pretreatment, the recovered DES-containing liquors were used as precursors for carbon dot production, providing an auxiliary valorization route for biomass-derived byproducts. Compared with the CDs prepared from the recovered ChCl-MEA liquor, the CDs derived from the recovered ChCl-MEA-H_2_O_2_ liquor exhibited better antioxidant and antibacterial activities, with DPPH and ABTS radical-scavenging activities reaching 85.2 ± 2.1% and 93.7 ± 2.2%, respectively, and inhibition-zone diameters against *S. aureus* and *E. coli* reaching 5 mm and 4 mm, respectively. Overall, this work provides a feasible strategy for integrating bamboo fractionation, bioethanol production, and recovered-liquor valorization within ChCl-MEA-based DES systems.

## Data Availability

The original contributions presented in this study are included in the article/Supplementary Material. Further inquiries can be directed to the corresponding author.

## Supplementary Materials

The following supporting information can be downloaded at: https://www.mdpi.com/article/10.3390/molecules31111832/s1. The supporting information includes: Figure S1: FT-IR spectra; Figure S2: Yields of xylose via enzymatic hydrolysis; Figures S3–S5: physicochemical property characterization of ChCl-MEA-based solvent systems.
