# Supplementary material for: Third-Component-Regulated Choline Chloride–Monoethanolamine-Based Solvent Systems for Enhanced Valorization of Bamboo Toward Concurrent Bioethanol and Carbon Dot Production
Source: Molecules. 2026 May 26;31(11):1832. doi: 10.3390/molecules31111832 (PMC13258785; doi:10.3390/molecules31111832)
Supplement: Supplementary file 1 [file molecules-31-01832-s001.zip › molecules-4311542-supplementary.pdf]

Supplementary Material

The supporting information provides supplementary FT-IR data (Figure S1), xylose-yield results (Figure S2), and physicochemical property characterization of DES systems (Figure S3–S5).

1. Experimental section

1.1. Chemical composition analysis of raw bamboo powder and pretreated residues

The contents of cellulose, hemicellulose, and acid-insoluble lignin in raw bamboo powder and the cellulose-rich residues obtained after pretreatment were determined according to the National Renewable Energy Laboratory (NREL) laboratory analytical procedure for biomass. All measurements were performed in triplicate, and the average values were reported. Solid yield, cellulose recovery, delignification, and hemicellulose removal during pretreatment were calculated according to Eqs. (S1)–(S4), where m_0 and m_1 represent the dry masses of raw bamboo and pretreated residue, respectively, and C, L, and H denote the cellulose, lignin, and hemicellulose contents. This analysis was used to evaluate the fractionation efficiency of different pretreatment systems toward bamboo cell-wall components.

$$\text{Solid yield (\%)} = m_1 / m_0 \times 100 \tag{S1}$$

$$\text{Cellulose recovery (\%)} = C_1m_1 / C_0m_0 \times 100 \tag{S2}$$

$$\text{Delignification (\%)} = [1 - (L_1m_1) / (L_0m_0)] \times 100 \tag{S3}$$

$$\text{Hemicellulose removal (\%)} = [1 - (H_1m_1) / (H_0m_0)] \times 100 \tag{S4}$$

where m_0 and m_1 are the dry masses of raw bamboo and pretreated residue, respectively; C_0 and C_1 are the cellulose mass fractions of raw bamboo and pretreated residue, respectively; L_0 and L_1 are the lignin mass fractions of raw bamboo and pretreated residue, respectively; and H_0 and H_1 are the hemicellulose mass fractions of raw bamboo and pretreated residue, respectively.

1.2. X-ray diffraction (XRD) analysis and calculation of *CrI*/Cellulose

The crystalline structures of the samples were analyzed using an X-ray diffractometer (D8 ADVANCE, Bruker, USA). The cellulose crystallinity index (*CrI*) was calculated by the Segal method according to Eq. (S5):

$$CrI\ (\%) = (I200 - Iam) / I200 \times 100 \tag{S5}$$

where I200 is the diffraction intensity of the main crystalline plane at approximately 2θ = 22.5°, and Iam is the diffraction intensity of the amorphous region at approximately 2θ = 18°. To reduce the influence of cellulose-content variation on the apparent crystallinity index, a normalized *CrI*/Cellulose parameter was further calculated according to Eq. (S6):

$$CrI/\text{Cellulose} = CrI / \text{Cellulose} \tag{S6}$$

where Cellulose is the cellulose mass fraction of the sample expressed as a decimal. This

parameter was used to reflect crystallinity changes relative to cellulose content.

1.3. X-ray photoelectron spectroscopy (XPS) analysis and calculation of surface lignin coverage

The surface elemental composition and chemical states of raw bamboo powder and pretreated residues were analyzed by X-ray photoelectron spectroscopy (ESCALAB 250Xi, Thermo Scientific, USA). The C 1s and O 1s spectra were collected and used to calculate the surface oxygen-to-carbon atomic ratio (O/C). The surface lignin coverage (SLC) was then calculated according to Eq. (S7) [3]:

$$SLC\ (\%) = 100 \times (0.83 - O/C_{sample}) / (0.83 - 0.33) \quad (S7)$$

where O/C sample is the measured surface oxygen-to-carbon atomic ratio of the sample. The constants 0.83 and 0.33 are the theoretical O/C ratios of carbohydrates and lignin, respectively. A higher SLC value indicates a higher degree of lignin coverage on the sample surface.

1.4. Degree of polymerization (DP) determination

The degree of polymerization of the cellulose-rich residues was determined by the cupriethylenediamine (CED) viscosity method. To minimize interference from residual lignin, the samples were bleached prior to analysis. Specifically, a 5% (w/v) sodium chlorite solution was prepared and adjusted to pH 4.0 with acetic acid. The absolutely dry sample was then mixed with the bleaching liquor at a solid-to-liquid ratio of 1:20 (w/v) and reacted in a water bath at 70 °C for 1.5 h. After bleaching, the sample was filtered, thoroughly washed with deionized water until neutral, and freeze-dried.

Then, 0.1 g of the bleached sample was dispersed in 20 mL of CED solution and magnetically stirred for 12 h at room temperature under sealed and light-protected conditions to ensure complete dissolution. The efflux time of the solution was measured using a capillary viscometer, and the intrinsic viscosity was converted to the degree of polymerization according to TAPPI T230 om-99. Each sample was measured in triplicate. The DP value was calculated according to Eq. (S8):

$$DP = -449.6 + 598.4 \ln[\eta] + 118.02 (\ln[\eta])^2 \quad (S8)$$

where $[\eta]$ is the intrinsic viscosity of cellulose dissolved in CED. A higher DP value indicates better preservation of cellulose chain integrity, whereas a decrease in DP suggests degradation of cellulose chains during pretreatment.

1.5. Hydrophobicity determination

The surface hydrophobicity of raw bamboo powder and pretreated residues was determined by Rose Bengal adsorption. A 40 mg/L Rose Bengal stock solution was prepared in citrate buffer (pH 4.8). Standard solutions with concentrations of 0, 10, 20, 30, and 40 mg/L were prepared, and their absorbance values were measured at 543 nm to construct a calibration curve.

For the adsorption assay, 0.03, 0.06, 0.09, 0.12, and 0.15 g of sample were placed into centrifuge tubes, and 3 mL of 40 mg/L Rose Bengal solution was added to each tube. The mixtures were shaken at 50 °C and 150 rpm for 2 h, followed by centrifugation. The absorbance of the supernatants was measured at 543 nm, and the concentration of

unadsorbed dye was obtained from the calibration curve. The relative hydrophobicity of each sample was represented by the slope of the linear fit between the amount of adsorbed Rose Bengal and the sample dosage. A larger slope indicates more hydrophobic sites exposed on the sample surface.

1.6. Cellulase adsorption capacity

The cellulase adsorption behavior of raw bamboo powder and pretreated residues was evaluated using enzyme solutions with different initial protein concentrations. The residual protein concentration in the supernatant was determined by the Bradford method. Bradford reagent was prepared by dissolving 0.1 g of Coomassie Brilliant Blue G-250 in 50 mL of 95% ethanol, followed by the addition of 100 mL of 85% phosphoric acid and dilution to 1000 mL with deionized water. Bovine serum albumin (BSA) standard solutions with concentrations of 0, 0.25, 0.50, 0.75, and 1.00 mg/mL were used to construct the Bradford calibration curve.

For the adsorption experiment, 0.1 g of sample was placed in a dry centrifuge tube, and 5 mL of cellulase solution with protein concentrations of 0, 0.01, 0.02, 0.04, 0.08, 0.16, 0.5, and 1.0 mg/mL was added. The pH of the system was adjusted to 4.8 using citrate buffer. The mixtures were incubated at 4 °C and 150 rpm for 3 h and then centrifuged. The residual protein concentration in the supernatant was quantified by the Bradford assay.

The adsorption isotherms were fitted using the Langmuir model, and the corresponding parameters were calculated using Eqs. (S9) and (S10):

$$\Gamma = KC\Gamma max / (1 + KC) \quad \text{(S9)}$$

$$R = K\Gamma max \quad \text{(S10)}$$

where Γ is the amount of cellulase adsorbed on the substrate (mg/g), K is the Langmuir constant reflecting the affinity between the sample and cellulase, C is the concentration of free cellulase in the supernatant (mg/mL), Γmax is the maximum adsorption capacity (mg/g), and R is the binding-strength parameter (L/g). The values of Γmax and R were used to evaluate the nonproductive adsorption of cellulase onto the sample surface.

1.7. Enzyme accessibility determination

The enzyme accessibility of raw bamboo powder and pretreated residues was evaluated by Direct Red 28 adsorption. A 3 g/L Direct Red 28 stock solution was prepared, and standard solutions with concentrations of 0, 0.5, 1.0, 2.0, and 3.0 g/L were used to establish a calibration curve by measuring the absorbance at 498 nm.

For the adsorption assay, 0.08 g of sample was added to each centrifuge tube, followed by 8 mL of Direct Red 28 solution with concentrations of 0, 0.05, 0.10, 0.50, 1.00, 2.00, and 3.00 g/L. The suspensions were shaken at 50 °C and 150 rpm for 24 h and then centrifuged. The absorbance of the supernatants was measured at 498 nm, and the equilibrium dye concentrations were obtained from the calibration curve. Adsorption curves were then constructed, and the maximum dye uptake obtained by Langmuir fitting was used to represent enzyme accessibility. A higher maximum adsorption capacity indicates better accessibility of the substrate to enzyme-sized molecules.

1.8. Antioxidant activity of carbon dots

The antioxidant activity of the CDs was evaluated by DPPH and ABTS radical-scavenging assays. Prior to analysis, the CDs were dispersed in deionized water at a concentration of 1 mg/mL.

For the DPPH assay, 0.64 mL of the CD dispersion was mixed with 2.36 mL of methanolic DPPH solution, giving a final DPPH concentration of 75 μM. After vigorous mixing, the reaction mixture was kept in the dark at room temperature for 30 min, and the absorbance was measured at 515 nm. The blank control consisted of the DPPH solution without sample.

For the ABTS assay, a stock ABTS radical solution was prepared by mixing 7 mM ABTS solution with 2.45 mM potassium persulfate solution at a volume ratio of 1:1 and storing the mixture in the dark at room temperature for 16 h. Before use, the ABTS stock solution was diluted with deionized water to an absorbance of 0.70 ± 0.02 at 734 nm. Then, 0.5 mL of the CD dispersion was mixed with 2.0 mL of the ABTS working solution, and the reaction was allowed to proceed in the dark at 37 °C for 6 min before measuring the absorbance at 734 nm. All measurements were performed in triplicate.

The radical-scavenging rate was calculated according to Eq. (S11):

$$\text{Scavenging rate (\%)} = (A0 - As) / A0 \times 100 \qquad \text{(S11)}$$

where A0 is the absorbance of the blank control and As is the absorbance of the sample-containing solution. A higher scavenging rate indicates stronger antioxidant activity.

1.9. Antibacterial activity of carbon dots

The antibacterial activity of the CDs was evaluated by the agar well diffusion method using Staphylococcus aureus and Escherichia coli as the test strains. Activated bacterial strains were inoculated into liquid medium and cultured at 37 °C for 12–18 h with shaking. The bacterial suspensions were then adjusted with sterile saline to 0.5 McFarland turbidity, corresponding to approximately 1.5×10^8 CFU/mL, and further diluted to 1.0×10^6 CFU/mL as the working suspensions.

An aliquot of 100 μL of the working bacterial suspension was uniformly spread on the surface of Mueller–Hinton agar plates and allowed to absorb. Under sterile conditions, wells of 2 mm in diameter were punched into the agar, and 20 μL of CD dispersion (1 mg/mL) was added into each well. The plates were incubated in an inverted position at 37 °C for 24 h. The diameters of the inhibition zones were then measured to evaluate the antibacterial activity of the samples. All experiments were carried out in triplicate.

2. Tables and Figures

2.1. FT-IR Analysis

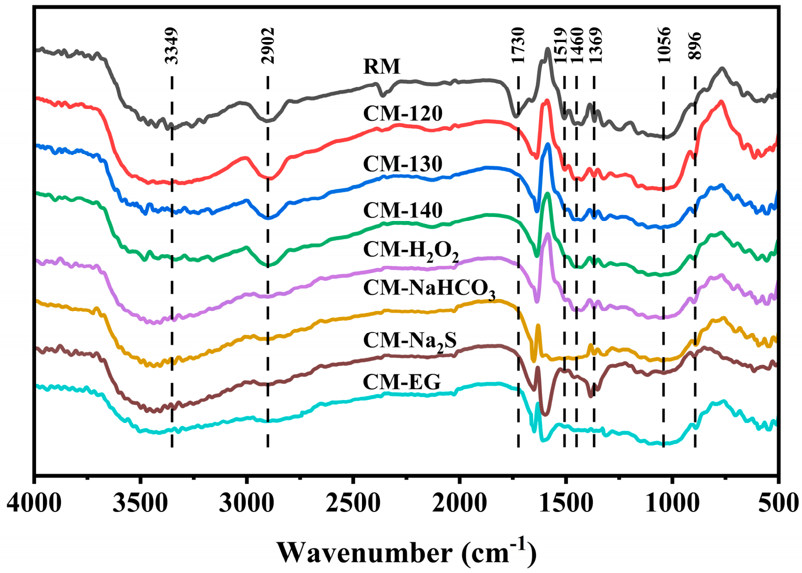

Figure S1. FT-IR spectra of raw bamboo and DES-pretreated residues.

2.2. Xylose Yield

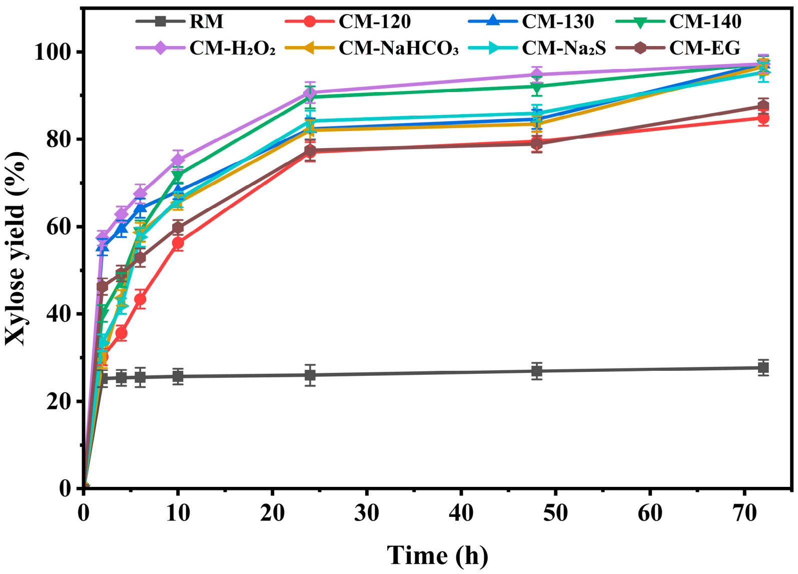

Figure S2. Time-resolved xylose yields during enzymatic hydrolysis of bamboo residues pretreated with the binary and third-component-modified ChCl-MEA systems.

2.3. Physicochemical Properties and Structural Stability of DESs

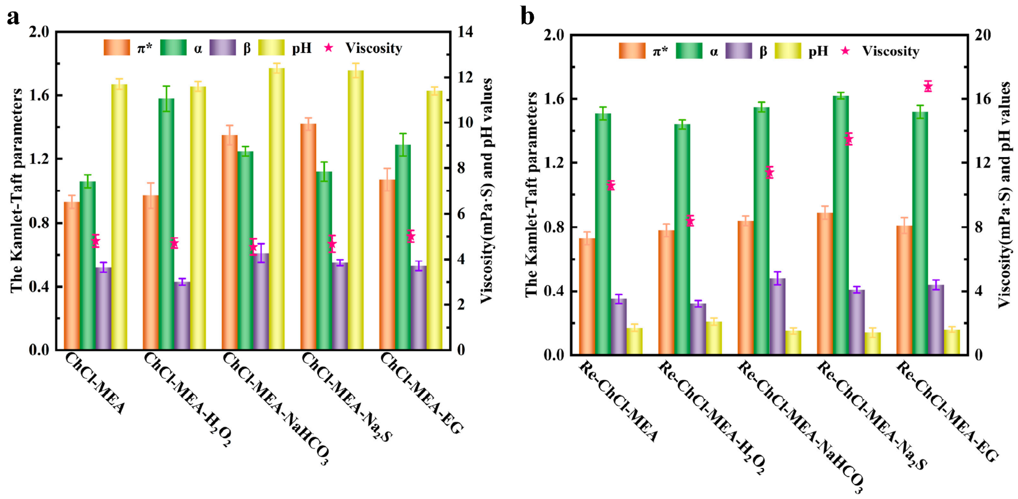

Figure S3. Physicochemical parameters of fresh and recovered DES systems, including polarity-related parameters, pH, and viscosity.

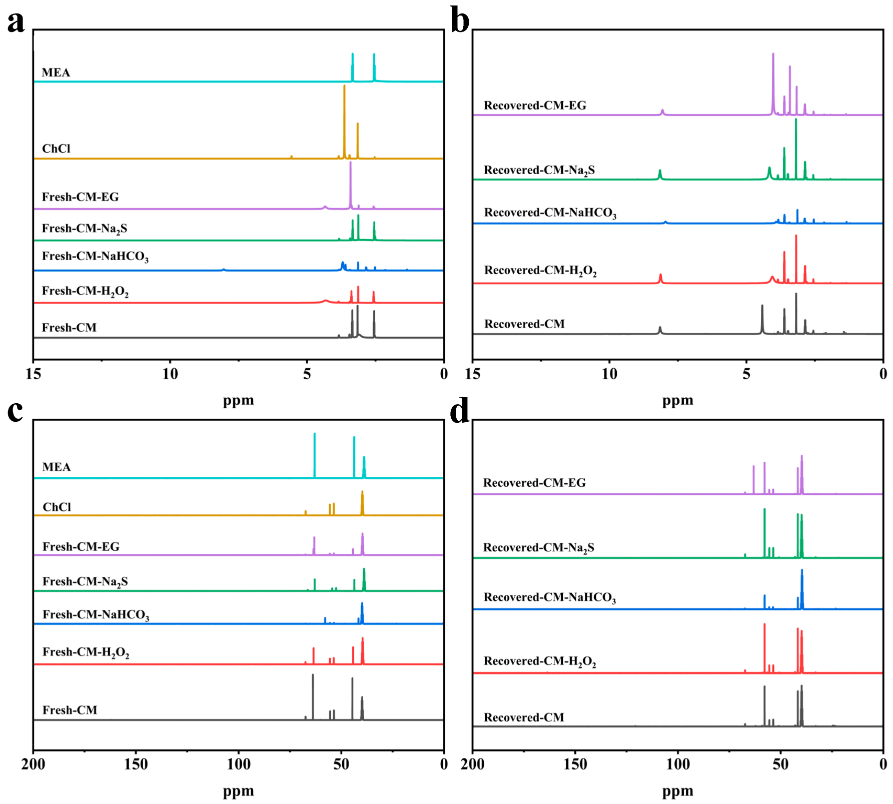

Figure S4. ^{1}H NMR spectra (a,b) and ^{13}C NMR spectra (c,d) of fresh and recovered DES systems.

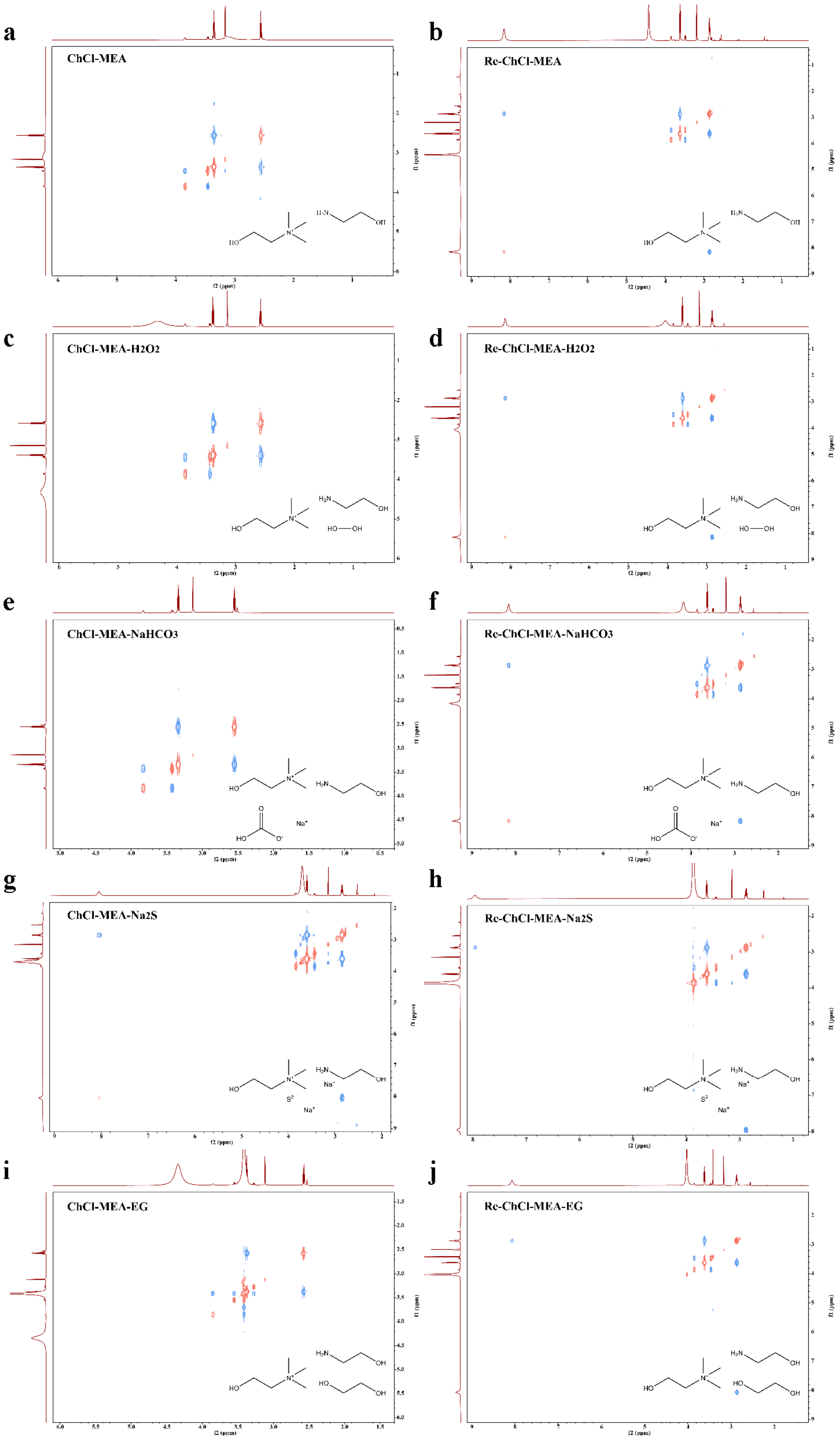

Figure S5. Two-dimensional NOESY spectra of fresh and recovered DES systems, illustrating changes in intermolecular interactions and hydrogen-bonding environments after reuse.
